# Poorly differentiated oesophageal squamous cell carcinoma in Western Kenya: A case report 

**DOI:** 10.4102/phcfm.v3i1.236

**Published:** 2011-07-14

**Authors:** Masemiano P. Chege

**Affiliations:** 1 Department of family Medicine, Moi University School of Medicine, Kenya

## Introduction

Cancer of the oesophagus is the eighth (some articles put it as sixth) most common cancer in the world. Squamous cell carcinoma is the predominant histological type with the highest incidence in the *‘Asian oesophageal cancer belt’*.^1^ A recent study documented the unusual findings of oesophageal cancer in young Kenyans from Western Kenya and suspected genetic or familial risk factors could be involved.^2^ The suspected genetic risk has been documented in studies conducted in Asia where the condition is prevalent.^3,4,5,6,7,8^ 

Patients with oesophageal cancer tend to present to rural hospitals in sub-Saharan Africa when it is too late (usually when the advanced disease has made it difficult for them to swallow even saliva). The outcomes of care are limited by the late presentation, unavailability of diagnostic and healthcare resources in these rural district hospitals and poverty in amongst local people. In most cases all we can do is a barium swallow to strengthen our clinical suspicion, because endoscopic and histology services are located far from these hospitals. Those patients that can afford the histology will, in nearly all cases, find it difficult to pursue further specialised care. This implies that the clinical suspicion of oesophageal cancer in these facilities translates to certain death within weeks. Consequently, we would like to present this case and compile a literature review on risk factors for oesophageal squamous cell carcinoma (OSCC) to highlight this dilemma. We also want to demonstrate our limitations in managing this severe disease which affects young patients who do not seem to have been exposed to the traditionally cited risk factors in other parts of the world where oesophageal carcinoma is prevalent. 

## Ethical considerations

The ethical consideration for this case report was approved by Moi University Institutional Research Ethics Committee (IREC) and the administration Webuye district hospital where the patient was managed. 

## Case report 

A 17-year-old girl presented with progressive difficulty in swallowing which was associated with retrosternal pain and progressive wasting over a two month period, in our regional (provincial) hospital. She also reported coughing productive and that was occasionally blood stained. 

Swallowing was not painful but initially she had trouble swallowing solid food and had the sensation that the food was lodged in her ‘chest’. This was followed by a progressive regurgitation of solids and finally liquids, including saliva, within a period of eight weeks. 

There was no history of fever or night sweats. None of her family members was infected with tuberculosis at present or had been diagnosed with tuberculosis in the recent past. Her health history was insignificant. She was the third in a family of five siblings. All the other siblings and her parents were in good health. There was no family history of cancer or chronic noncommunicable disease. 

When she was admitted to our hospital, she was already being treated for pulmonary tuberculosis with the diagnosis based on a chest x-ray examination in the regional hospital. 

On examination she was found to be severely wasted and dehydrated, but without fever or enlarged superficial lymph nodes. The other significant clinical findings were of left sided pleural effusion. 

A repeat chest x-ray confirmed the pleural effusion which was found to be straw coloured on tapping, with a high protein but insignificant white cell count. The full haemogram, renal and liver function tests were normal. A diagnosis of a possible oesophageal fistula was entertained and she was referred for an upper gastrointestinal tract endoscopy, 80 kilometres away. 

The endoscopy revealed that she had a fungating obstructive mass at a position of 20 centimetres from the top of the oesophagus that did not allow further advancement of the scope. A biopsy was taken which histology showed poorly differentiated squamous cell carcinoma. 

The patient and her parents were well briefed about the endoscopic and histology findings and the patient was registered for palliative care. The possibility of palliative stenting of the mass in a missionary hospital 350 kilometres away was discussed with the family but they declined because of financial constraints. The parents opted to nurse her at home with support from our palliative care team. Her condition continued to deteriorate and she died at home two weeks after the diagnosis was confirmed. 

## Discussion

Heavy smoking and alcohol consumption (over an extended period of time) are well documented risk factors for oesophageal squamous cell carcinoma (OSCC).^9,10,11,12,13,14,15,16,17,18,19^ A diet that is high in cholesterol, refined sugars, but low in fibre and fruit has been associated with an increased incidence of OSCC.^20^ Pickled vegetables, commonly ingested in Asia, has been associated with an increased incidence of oesophageal cancer.^21^ Some studies have documented an association of Human Papilloma Virus (HPV) infection with an increased incidence of oesophageal cancer.^22,23^

In Kenya oesophageal cancer is geographically unevenly distributed with a high incidence in very specific districts that are erratically spread. A study in the regional hospital for Western Kenya rated oesophageal cancer as the most common male malignancy and the third most common female malignancy in that region.^24^ The study documented the mean age for this condition as 58.7 years and more than 90% of the cases concerned squamous cell carcinoma. A male-to-female ratio of 1.5:1 was documented. Risk factors for the condition were not studied. 

A higher prevalence of OSCC in male patients has been documented in many studies and this may not be unexpected because the prevalence of heavy smoking and drinking is higher in men than in women in the areas with the highest prevalence of the condition. There has been speculation on the possibility of the protective role of oestrogen.^25^ 

In this case we conducted a thorough history on the patient’s health, her family’s socio-economic status, and her dietary habits from the time she was born. We enquired about the environment where she was born and brought up. This did not reveal risk factors for OSCC. We do, however, acknowledge our limitation in how far our work-up could go, as well as the degree of curative support we could offer in our rural district hospital in Kenya. 

## Conclusion

The occurrence of oesophageal cancer in young people in this part of Kenya is a wake-up call for workers in healthcare, public health and pathology to work together to research and identify risk factors that could explain the early onset of OSCC. This will provide information for the Public Health Policy and Planning to devise ways of managing modifiable risk factors, the promotion of health and developing methods for early detection as well as improving management outcomes. 
